# Pain management for necrotizing enterocolitis: getting the balance right

**DOI:** 10.1038/s41390-022-01968-2

**Published:** 2022-02-15

**Authors:** Judith A. ten Barge, Marijn J. Vermeulen, Sinno H. P. Simons, Gerbrich E. van den Bosch

**Affiliations:** 1 Netherlands Institute for Health Sciences, Rotterdam, The Netherlands; 2 Division of Neonatology, Department of Pediatrics, Sophia Children’s Hospital, Rotterdam, The Netherlands

## Abstract

**Background:**

Adequate pain management for preterm born neonates suffering from the extremely painful disease necrotizing enterocolitis (NEC) is essential, since neonatal exposure to pain is related to negative short-term and long-term consequences. The aim of this study was to describe the current pain management and its effectiveness in NEC patients.

**Methods:**

In this single-center, retrospective study, neonates (gestational age < 32 weeks and/or birth weight < 1500 g) with NEC Bell’s stage II or III were included. Information on pain (based on COMFORTneo and NRS scores) and analgesic therapy was collected and analyzed for the acute disease period.

**Results:**

Of 79 patients included, 74 (94%) received intravenous analgesic therapy: most commonly morphine, fentanyl, and acetaminophen. The median COMFORTneo score was 11 (IQR 10–11), however, 49 patients had at least one COMFORTneo score ≥ 14 indicating pain. Nineteen patients had persistent high pain scores ≥ 14 with a median duration of 7.2 h (IQR 2.8–14.0).

**Conclusions:**

This study showed that despite analgesic therapy, most NEC patients showed signs of pain, and in some, pain persisted for several hours. It suggests that current analgesic therapy frequently failed to prevent pain and existing pain was often insufficiently treated. This supports the urgent need for individualized pain management guidelines for NEC patients.

**Impact:**

- This study is unique in reporting on pain management in neonates suffering from necrotizing enterocolitis (NEC) during the full acute disease period.
- Despite analgesic therapy, the majority of NEC patients experience pain, and in some patients, pain persists for several hours.
- These findings highlight the need for improvement of neonatal pain management in NEC patients, including better pain monitoring and guidelines for individualized analgesic therapy.
- Improved pain management guidelines may help to prevent short-term and long-term consequences of neonatal exposure to pain, as well as excessive exposure to opioids.

## Introduction

Necrotizing enterocolitis (NEC) is an extremely painful and life-threatening disease in preterm born neonates, which affects approximately 5–10% of very-low-birth-weight (VLBW, <1500 g) infants.^1^ It is characterized by a painful excessive inflammatory process in the intestine, which quickly spreads and can eventually have systemic effects with a negative impact on distant organs such as the brain.^2^ NEC is associated with high mortality, ranging from 15 to 50%, depending on birth weight and severity of NEC, and is particularly high in VLBW infants requiring surgical intervention.^3^ Among NEC survivors short-bowel syndrome and neurodevelopmental disabilities are common, resulting in significant long-term morbidity.^3^

Treatment of NEC primarily consists of medical treatment, including abdominal decompression, broad-spectrum intravenous antibiotics, no enteral feeding, and therefore total parenteral nutrition.^2^ Surgical intervention is required if the patient does not recover or in case of bowel perforation, and comprises resection of the affected intestinal parts, often with the placement of an enterostomy. An essential aspect of NEC treatment is adequate pain management, since it is a very painful disease due to the ongoing inflammation and ischemia in the intestine. In contrast to what clinicians once thought, preterm neonates can experience pain and it is important to adequately treat their pain in order to prevent discomfort and severe short-term metabolic and circulatory complications.^4^ Exposure to pain during the neonatal period not only threatens short-term comfort and recovery, but can also have negative long-term consequences by harming neurodevelopment.^5^ Rodent studies suggest that exposure to pain during the neonatal period affects brain development and induces long-term behavioral changes.^6–11^ In humans, neonatal exposure to pain has been associated with changes in brain morphology, impaired cognitive development, and altered pain sensitivity.^12–22^ These negative effects were already found in children who had been exposed to mild procedural pain during NICU admission. NEC, on the other hand, causes severe visceral pain, and is therefore expected to have even more negative long-term consequences.^23^ Additionally, NEC patients are often very preterm, which makes them specifically vulnerable to the negative effects of pain due to the immaturity of the developing nociceptive system. On top of that, seeing your vulnerable child experiencing pain is very stressful for parents.^24^

Opioids such as morphine and fentanyl are most commonly used for treating pain in neonates suffering from NEC.^25^ When treating neonates with opioids, caution is needed, as animal studies have shown that neonatal exposure to opioids could potentially harm neurodevelopment.^8–10^ However, these rodent studies found that exposure to opioids in the presence of pain has a neuroprotective effect.^8–10^ In humans, contradictory effects of neonatal opioid exposure on brain morphology and cognition have been suggested.^5^

Since both exposure to pain and to opioids during the neonatal period could have negative short-term and long-term effects, it is essential to adequately treat pain in NEC patients, while avoiding overtreatment and side effects. In other words: getting the balance right. Currently, it is unknown whether pain in neonates with NEC is treated adequately or whether these neonates are exposed to excessive pain or overtreatment with opioids. Importantly, it has been suggested that the current pain treatment of neonates suffering from NEC is not balanced yet. For instance, Van Ganzewinkel et al. found that NEC survivors had a decreased pain threshold and pain tolerance in adolescence, which may reflect the effects of neonatal exposure to excessive pain.^26^ To find out whether current pain management for NEC patients is well-balanced or if patients receive too little or too much analgesic therapy, this study aims to describe current pain management in preterm neonates suffering from NEC.

## Methods

### Study design and sample

In this single-center, retrospective study, all neonates diagnosed with NEC who were admitted to the level III-IV neonatal intensive care unit of the Erasmus MC—Sophia Children’s Hospital, Rotterdam, The Netherlands, between January 1, 2015, and December 31, 2020, were included. Only patients with NEC stage ≥ II, according to modified Bell’s criteria, were included.^27^ NEC diagnosis and staging according to these criteria was based on clinical symptoms, radiographic findings, surgical and pathology reports and decided on in expert consensus meetings. Infants with spontaneous intestinal perforation (SIP) were excluded. Surgical NEC was defined as NEC with a surgical indication within 7 days after NEC onset.

The Erasmus MC—Sophia Children’s Hospital is a teaching hospital with open-bay units and 24-h accessibility of the ward for parents, who participate in the care for their child and generally provide kangaroo care for a few hours daily if the child’s condition allows.

This study was embedded in the Risk NEC Study, an ongoing local cohort study on risk factors, treatment, and outcomes related to NEC in very preterm infants. The Risk NEC Study started in 2008 and includes patients with a birth weight < 1500 grams and/or a gestational age < 32 weeks who are admitted to the NICU of the Erasmus MC—Sophia Children’s Hospital. This study aims to assess risk factors and outcomes of NEC. The institutional medical ethical review board waived the need for specific approval for this study according to the Dutch law (MEC-2013-409), based on the observational nature of the study. No specific informed consent was deemed necessary for the use of the retrospective data in this study.

### Data collection

Demographics were extracted from the existing database of the ongoing Risk NEC Study. Pain and pharmacological data were additionally retrieved from the electronic patient management systems HiX© (Chipsoft, Amsterdam, The Netherlands) and Picis© (Picis Clinical Solutions, Inc., Wakefield, Massachusetts) for each individual NEC disease period. The onset of the disease period was defined as the day of prescription and the start of antibiotic treatment as part of (suspected) NEC (e.g., Augmentin/Gentamicin). The last day of the NEC disease period was defined as the second day after the administration of intravenous analgesics had been discontinued or until death. If a patient had not received intravenous analgesic therapy, the NEC disease period ended on the second day after re-initiation of minimal enteral feeding (MEF).

The effectiveness of the analgesic therapy was determined using the neonates’ COMFORTneo, NRS pain, and NRS distress scores. These pain scores were collected for each patient for the total disease period. Patients were treated according to the local pain guideline (Supplement 2), which prescribes starting with acetaminophen and optionally combining this with morphine or fentanyl guided by the pain scores. Pharmacological data on dosage and duration of the analgesics acetaminophen, morphine and fentanyl, the sedative midazolam, the anesthetics ketamine and propofol, and the muscle relaxants rocuronium and cisatracurium were collected from the electronic patient management systems, which were used to calculate each patient’s daily cumulative doses during the disease period. The median daily cumulative dose and highest daily cumulative dose were calculated for each patient.

### Measurement instruments

Nurses in our NICU have been trained to assess the COMFORTneo, NRS pain, and NRS distress score and enter these in the electronic patient management system. The COMFORTneo score (Supplement 1) ranges from 6 to 30 and has been validated for assessment of prolonged pain in preterm neonates.^28^ The COMFORTneo score has a good interrater reliability among trained nurses.^28^ A COMFORTneo score of 14 or higher indicates pain and a COMFORTneo score of 8 or lower indicates possible analgesic overtreatment. The NRS pain and NRS distress scores range from 0 to 10 (0 = no pain/distress, 10 = worst imaginable pain/distress) and represent a nurse’s subjective estimate of the patient’s pain and distress. An NRS pain score of 4 or higher indicates pain and an NRS distress score of 4 or higher indicates distress.

The local pain management protocol prescribes three measurements per day and additional measurements in case of suspected pain or oversedation and after a change of analgesic therapy (Supplement 2).^29^ A previous study in our center has shown that the compliance rate to these prescribed measurements is 60%.^29^

### Statistical analyses

The pain scores and dosages of analgesics are presented as medians (interquartile ranges) or numbers (percentages). The number of patients with a COMFORTneo score ≤ 8 was calculated among patients who received intravenous analgesics. The median doses of analgesics were calculated among patients receiving those analgesics.

In order to evaluate the agreement between different pain scores used in our NICU, the positive and negative percent agreement between these scores were calculated. The positive percent agreement between the COMFORTneo score and the NRS pain and NRS distress score was calculated by dividing the number of times both scores indicated pain by the number of times the COMFORTneo score indicated pain. The negative percent agreement was calculated by dividing the number of times both scores indicated no pain by the number of times the COMFORTneo score indicated no pain.

To look further into different patterns of pain, a subgroup analysis was performed, based on three pain score patterns: persistent high scores, persistent low scores, and recurrent high scores. Persistent high scores were defined as at least two consecutive COMFORTneo scores ≥ 14 with at least 1 h between these scores. Recurrent high scores were defined as at least 3 COMFORTneo scores ≥ 14 with normal scores (i.e., 9–13) in between. Persistent low scores were defined as at least two consecutive COMFORTneo scores ≤ 8 with at least 1 h between these scores. The number of patients with persistent low scores was calculated among patients receiving analgesic therapy. The association between COMFORTneo score and analgesic therapy was visualized for the patient with the most illustrative pain patterns, selected based on an independent review of all patient plots by three neonatologists. Analyses were conducted using RStudio version 1.4.1106 (R Core Team, Vienna, Austria) and SPSS version 25.0 (IBM Corp., Armonk, New York).

## Results

During the study period, 4074 neonates were primarily admitted to the NICU of the Erasmus MC—Sophia Children’s Hospital, of whom 1524 neonates were born at a gestational age < 32 weeks. Seventy-nine preterm neonates diagnosed with NEC were included, corresponding to a NEC rate of 5.2% among very preterm neonates. Table 1 shows the patients’ background and clinical characteristics. The median gestational age was 26 (IQR 25–28) weeks and the median postnatal age at diagnosis was 10 (IQR 7–20) days. Nineteen patients had NEC stage II and 60 patients had NEC stage III. Fifty-nine patients had surgical NEC (i.e., indication for surgery within a week), of whom 49 patients received surgery, since 10 surgical NEC patients were not stable enough to tolerate surgery and died. Two patients received surgery but were not classified as surgical NEC, since the indication for surgery was not within a week. Overall, 38 patients (48%) died during NICU admission, 33 deaths (42%) were directly related to NEC.

**Table 1:** Background and clinical characteristics.

Variable	
Sex	
Male	46 (58.2)
Female	33 (41.8)
Mode of delivery	
Vaginal birth	34 (43.0)
Cesarean section	45 (57.0)
Inborn	79 (100)
Received steroids	74 (93.7)
Nutrition type	
Only formula	2 (2.5)
Only breastmilk	38 (48.1)
Both	38 (48.1)
Birth weight in grams	835 (733–1050)
Gestational age at birth in weeks	26 (25–28)
*Z*-score Fenton 2013 Preterm Growth Chart	0.102 (−0.370–0.604)
Apgar score	8 (7–9)
NEC stage	
Stage II	19 (24.1)
Stage III	60 (75.9)
Postnatal age at diagnosis in days	10 (7–20)
Surgical NEC	59 (74.7)
Underwent surgery	51 (64.6)
Use of inotropes/vasopressors during NEC	
Overall	60 (75.9)
Only during surgery	26 (32.9)
Mechanically ventilated during NEC	69 (87.3)
Died	38 (48.1)
NEC-related mortality	33 (41.8)

Values are expressed as median (IQR) or number (%).

### Assessment of pain scores

**Fig. 1:**
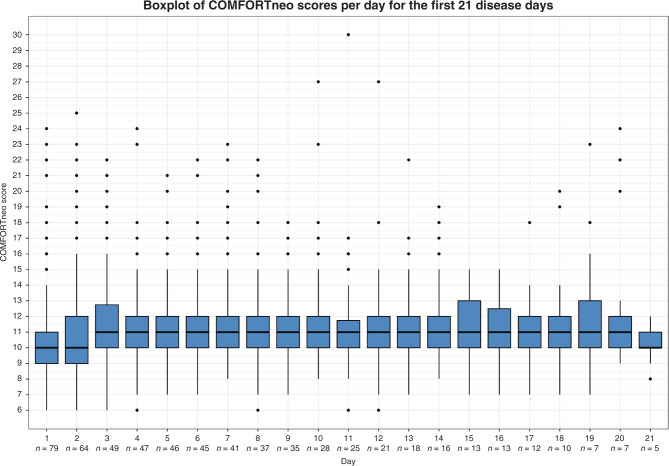
Boxplot depicting the longitudinal evolution of COMFORTneo score. The number of patients remaining in the cohort is indicated below the day. The distribution of COMFORTneo scores is rather constant over time, with an acceptable median COMFORTneo score but also frequent high scores.

**Fig. 2:**
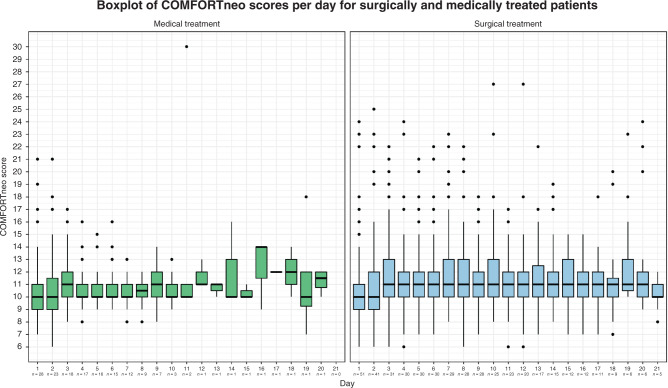
Boxplot depicting the longitudinal evolution of COMFORTneo score in medically and surgically treated patients. The number of patients remaining is indicated below the day. Surgically treated patients had more high COMFORTneo scores and these high scores occurred over a longer period.

The duration of the NEC disease period ranged from 1 to 47 days (median 7, IQR 2–12) and the total number of pain score measurements per patient ranged from 0 to 195 (median 21, IQR 4–41). At least one COMFORTneo score, NRS pain score or NRS distress score was available for 76 patients (96%). In three patients, no pain score was available, likely because these patients died shortly after the diagnosis of NEC. Summary statistics for the pain scores are shown in Table 2. The overall median of the individual median COMFORTneo scores per patient was 11 (IQR 10–11) and the overall median of the highest COMFORTneo scores per patient was 15 (IQR 12–20). Forty-nine patients (62%) had at least one COMFORTneo score ≥ 14. Fifty patients (63%) had at least one COMFORTneo score ≤ 8 while treated with intravenous analgesics. In four cases (2.6%) of all COMFORTneo scores ≤ 8, the NRS pain or NRS distress score was ≥ 4, indicating that analgesic overtreatment was unlikely. Figure 1 shows the distribution of COMFORTneo scores during the first 21 NEC disease days and the number of patients in the cohort (i.e., still alive and suffering from NEC) per day. Figure 2 shows the distribution of COMFORTneo scores separately for medically and surgically treated patients. A cut-off of 21 days was chosen for both graphs, since only five patients had a longer disease duration than 21 days. The median of the individual median NRS pain and NRS distress scores was 0 (IQR 0–0), indicating that the median NRS pain and NRS distress scores in at least three-quarters of NEC patients equaled 0. The positive and negative percent agreement between the COMFORTneo score and NRS pain score were 31.8% and 98.6%, respectively, and the positive and negative percent agreement between the COMFORTneo score and NRS distress score were 32.9% and 99.6%, respectively.

**Table 2:** Pain scores in NEC patients (*N* = 79).

Pain measurement instrument	
*COMFORTneo score*	
Median number of scores per patient (IQR)	21 (4–41)
Median number of scores ≥ 14 per patient (IQR)	1 (0–4)
Median of the median scores per patient (IQR)	11 (10–11)
Median of the highest scores per patient (IQR)	15 (12–20)
Number of patients with at least one score ≥ 14 (%)	49 (62.0)
Number of patients with at least one score ≤ 8 (%)	50 (63.3)
*NRS pain score*	
Median number of scores per patient (IQR)	17 (4–36)
Median number of scores ≥ 4 per patient (IQR)	0 (0–1)
Median of the median scores per patient (IQR)	0 (0–0)
Median of the highest scores per patient (IQR)	3 (0–5)
Number of patients with at least one score ≥ 4 (%)	35 (44.3)
*NRS distress score*	
Median number of scores per patient (IQR)	17 (4–36)
Median number of scores ≥ 4 per patient (IQR)	0 (0–1)
Median of the median scores per patient (IQR)	0 (0–0)
Median of the highest scores per patient (IQR)	2 (1–4)
Number of patients with at least one score ≥ 4 (%)	29 (36.7)

### Treatment with analgesics, sedatives, and anesthetics

The total cumulative doses and daily cumulative doses of the administered analgesics are shown in Table 3. Overall, 74 out of 79 patients (94%) with NEC were treated with analgesics. The five patients that did not receive analgesics were four stage II patients with a mild disease course and one stage III patient that died shortly after the diagnosis. The most commonly used analgesics were intravenous morphine, fentanyl, and acetaminophen. Sixty-six patients (84%) were treated with morphine and their median daily cumulative dose was 194 μg/kg (IQR 138–275) for a median of two (IQR 1–6) days. This corresponds to a median continuous dose of 8 μg/kg/h. Fentanyl was administered to 60 patients (76%) and their median daily cumulative dose was 22 μg/kg (IQR 10–44) for a median three (IQR 1–6) days. Forty-eight patients (61%) received acetaminophen and they received a median daily cumulative dose of 22 mg/kg (IQR 19–24) for a median seven (IQR 2–12) days. Midazolam was administered to 31 patients (39%) with a median daily cumulative dose of 0.24 mg/kg (IQR 0.08–0.74) for a median of one (IQR 1–3) day. No interruptions in analgesic therapy were observed during the disease period. Twenty-six patients (33%) were treated with propofol for endotracheal intubation. Methadone was administered to one patient (1%) in order to prevent withdrawal symptoms after prolonged treatment with morphine and fentanyl. Rocuronium and cisatracurium were administered pre-operatively to 25 (32%) and 20 patients (25%), respectively. Ketamine, phenobarbital, and clonidine were administered to one patient (1%) each.

**Table 3:** Pharmacological treatment.

Analgesics	
*Any intravenous analgesic*	
Number of patients	74 (93.7)
*Acetaminophen*	
Number of patients	48 (60.8)
Total cumulative dose (mg/kg)	151 (41–263)
Daily cumulative dose (mg/kg)	22 (19–24)
Maximum daily cumulative dose (mg/kg)	24 (20–25)
*Morphine*	
Number of patients	66 (83.5)
Total cumulative dose (ug/kg)	508 (210–1327)
Daily cumulative dose (ug/kg)	194 (138–275)
Maximum daily cumulative dose (ug/kg)	278 (168–366)
*Fentanyl*	
Number of patients	60 (75.9)
Total cumulative dose (ug/kg)	22 (10–44)
Daily cumulative dose (ug/kg)	8 (4–22)
Maximum daily cumulative dose (ug/kg)	13 (5–30)
*Methadone*	
Number of patients	1 (1.3)
Total cumulative dose (mg/kg)	5.01 (5.01–5.01)
Daily cumulative dose (mg/kg)	0.09 (0.09–0.09)
Maximum daily cumulative dose (mg/kg)	1.52 (1.52–1.52)
**Sedatives**	
*Midazolam*	
Number of patients	31 (39.2)
Total cumulative dose (mg/kg)	0.27 (0.11–1.72)
Daily cumulative dose (mg/kg)	0.24 (0.08–0.74)
Maximum daily cumulative dose (mg/kg)	0.24 (0.09–0.92)
**Anesthetics**	
*Ketamine*	
Number of patients	1 (1.3)
Total cumulative dose (mg/kg)	6.32 (6.32–6.32)
Daily cumulative dose (mg/kg)	1.05 (1.05–1.05)
Maximum daily cumulative dose (mg/kg)	1.84 (1.84–1.84)
*Propofol*	
Number of patients	26 (32.9)
Total cumulative dose (mg/kg)	1.37 (1.00–2.40)
**Muscle relaxants**	
*Rocuronium*	
Number of patients (%)	25 (31.6)
Total cumulative dose (mg/kg)	1.23 (1.00–2.05)
*Cisatracurium*	
Number of patients	20 (25.3)
Total cumulative dose (mg/kg)	0.30 (0.24–0.52)
**Other**	
*Phenobarbital*	
Number of patients	1 (1.3)
*Clonidine*	
Number of patients	1 (1.3)

Values are expressed as median (IQR) or number (%).

**Fig. 3:**
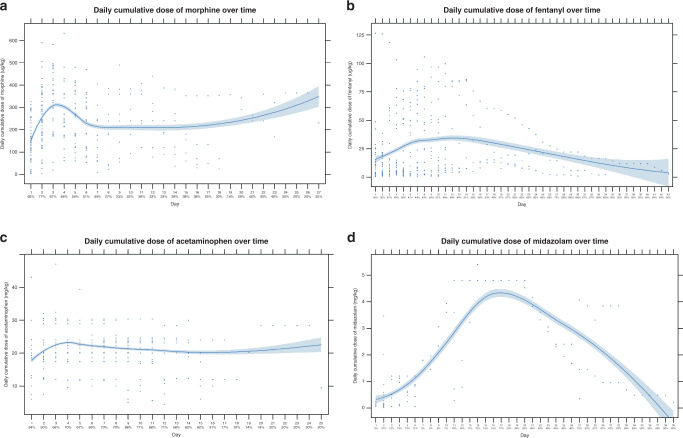
Scatter plots of the daily cumulative dose of morphine, fentanyl, acetaminophen, and midazolam over time with a superimposed smooth regression line and the corresponding standard error bounds. The percentage of the cohort receiving the analgesic is indicated below the day. **a** Morphine is mainly used during the first five days, with the daily cumulative dose peaking at day 3. **b** Fentanyl is used more frequently after the first week, representing a switch from morphine to fentanyl. **c** Acetaminophen use is initially low, but increases over the first week. **d** Midazolam use is common among patients with a very long disease period.

Daily cumulative doses of morphine, fentanyl, acetaminophen, and midazolam and the percentage of patients receiving these analgosedatives are shown in Fig. 3.

### Pain patterns

Persistent high COMFORTneo scores were found in 19 patients (24%), recurrent high scores in 24 patients (30%), and persistent low scores in 18 patients (23%) (Table 4). Some patients showed more than one pain pattern during their NEC episode, of whom six patients (8%) showed all three patterns. In 38 patients (48%), none of the described pain patterns were seen. In those with persistent high scores, the median duration of consecutive high pain scores was 7.2 h (IQR 2.8–14.0). In 62.5% of cases of persistent high scores, the analgesic therapy dosage was increased after the first high score. The median duration until an increase of dosage was 1.5 (IQR 0.5–3.6) h after the first high pain score. The median duration of consecutive low scores was 6.7 h (IQR 3.9–15.2). In 21.7% of cases of persistent low scores, the analgesic therapy dosage was decreased, which occurred a median duration of 3.2 (IQR 2.2–13.0) h after the first low score. The patients with recurrent high scores had a median number of six scores (IQR 4–15) above the pain threshold ≥ 14. In patients undergoing surgery, the three pain patterns (i.e., persistent high scores, persistent low scores, and recurrent high scores) were mostly seen during the entire post-operative period. In these patients, pain might be attributed to a prolonged post-operative course.

**Table 4:** Observed pain patterns during NEC.

	Persistent high scores	Persistent low scores	Recurrent high scores
Number of patients (%)	19 (24.1)	18 (22.8)	24 (30.4)
Number of patients undergoing surgery in subgroup (%)	16 (84.2)	14 (77.8)	21 (87.5)
Occurrence of these scores in relation to surgery (%)			
Pre-operative	3 (18.8)	5 (35.7)	0 (0)
Post-operative	11 (68.8)	8 (57.1)	12 (57.1)
Both	2 (12.5)	1 (7.1)	9 (42.9)
Median duration of persistent high/low COMFORTneo scores in hours (IQR)	7.2 (2.8–14.0)	6.7 (3.9–15.2)	
Median number of COMFORTneo scores ≥ 14 per patient (IQR)	6 (4–15)	3 (0–13)	6 (4–15)

Persistent high scores = two consecutive COMFORTneo scores ≥ 14 with a least 1 h between these scores.

Persistent low scores = two consecutive COMFORTneo scores ≤ 8 with a least 1 h between these scores.

Recurrent high scores = at least 3 COMFORTneo scores ≥ 14 with normal scores (i.e., 9–13) in between.

**Fig. 4:**
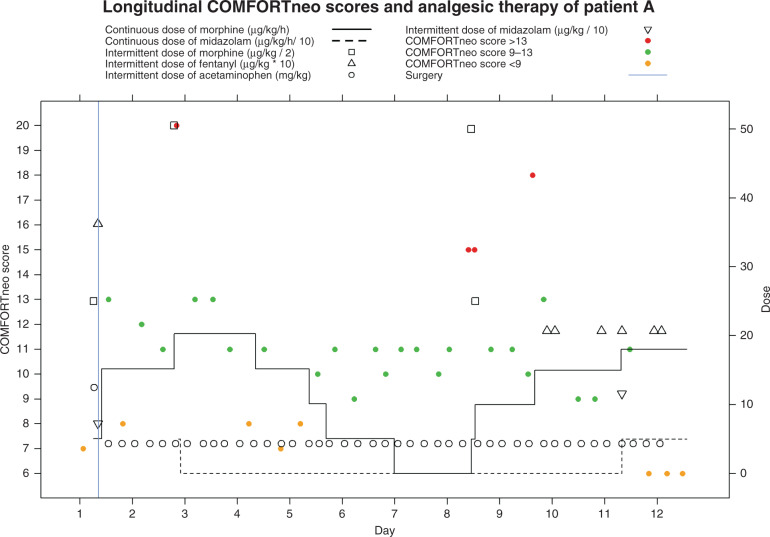
Overview of the longitudinal evolution of COMFORTneo scores and continuous and intermittent doses of analgesics of patient A. The COMFORTneo score is shown on the left y-axis and the dose is shown on the right *x* axis. The colored symbols represent the COMFORTneo scores, with red symbols indicating high scores, green symbols indicating acceptable scores, and orange symbols indicating low scores. The black lines indicate continuously administered analgosedatives and the different black symbols indicate intermittently administered analgosedatives. Patient A exhibits persistent high scores, persistent low scores, and recurrent high scores.

For the most illustrative patient with all pain patterns, pain scores and dosages of analgesics are shown over time (Fig. 4). Figure 4 shows that there was not only variation in pain level between patients, but also within a patient, with different pain patterns occurring over the disease course.

## Discussion

This retrospective cohort study on pain management in NEC patients showed that even though most pain scores were acceptable, over half of NEC patients showed signs of pain during their disease course, as indicated by a COMFORTneo score ≥ 14. Although the variation between individuals was high, the overall distribution of COMFORTneo scores seemed constant over the disease period. In NEC patients, repeated intermittent moments of pain were more common than prolonged periods of pain. Various pain patterns were observed in spite of analgesic therapy in 94% of NEC patients. Therapy included morphine, fentanyl, and acetaminophen that were often combined. Surprisingly, treatment with acetaminophen was initiated less frequent and on average later than treatment with morphine, even though the local pain protocol prescribes starting with acetaminophen as the first step in the treatment of NEC. We speculate that the relatively infrequent use of acetaminophen is because physicians sometimes forgot the prescription of acetaminophen rather than deliberate non-use. Our data suggest that analgesic therapy was generally effective, but failed to prevent pain at times in the majority of patients. Furthermore, analysis of pain patterns has shown that persistent pain, with a median duration of 7 h, was prevalent in a quarter of NEC patients, indicating that pain was often not relieved promptly after a high pain score. The analgesic dosage was increased in 62.5% of cases of persistent high pain scores, after a median duration of 1.5 h. Thus, persistent pain often occurred despite a change of analgesic therapy.

The data additionally show that analgesic therapy might have been excessive at times, since 63% of NEC patients had at least one COMFORTneo score ≤ 8 and persistent low scores were common too. However, low COMFORTneo scores in NEC patients do not necessarily indicate overtreatment; they can also be the result of low consciousness and reduced reactivity related to the disease itself. Importantly, the level and pattern of pain varied significantly between patients, reflecting the need for individualized pain treatments. This heterogeneity complicates the development of an appropriate pain management protocol for NEC patients. More research is needed to predict which patients are at risk of having a highly painful disease course.

To our knowledge, this study is unique in longitudinally exploring pain and analgesic therapy during complete NEC disease episodes. Only two previous papers have been published on pain and analgesic therapy in NEC patients.^25,30^ The first study, by Gibbins et al., assessed procedural pain in 25 stage II NEC patients using the Premature Infant Pain Profile (PIPP).^30^ They found that NEC patients on average experienced 16 painful procedures per day. However, the pain scores were not presented in this paper and nurses’ compliance with the pain assessment was low. The other paper, from our own research group, studied pain scores and analgesic therapy of NEC patients around the surgical intervention and showed that the median COMFORTneo score was 10 pre-operatively and 11 post-operatively and that most patients had at least one high pain score.^25^ Our current study confirms the earlier findings that pain scores are overall acceptable, but that the majority of NEC patients at least once have a high pain score. The current study additionally shows that pain occurs during the entire disease course of NEC, not just in the first days or around surgery, and that pain often persists. Acknowledging that NEC is likely the most painful condition in neonatal care, it is disappointing that no further studies on pain management in NEC have been published. There is a gap in knowledge, and consequently lack of consensus, on adequate drugs, doses, and pain assessment for these vulnerable patients. Larger prospective multi-center studies are highly needed.

A strength of our study is the complete and comprehensive overview of pain and pain management during the entire NEC disease period, in a substantial cohort of preterm infants that suffer from the rare disease NEC. A limitation of our study is the retrospective design, with the number of pain measurements differing between patients. The number of measurements may not only be related to the disease duration, but also to the severity of pain, since pain scores tend to be measured more frequently after high scores. This reflects good clinical practice, but may have led to over-representation of the painful periods, and may therefore have biased our results. The retrospective design also limited our ability to evaluate effectiveness of specific therapeutic interventions. As systematic pre- and post-intervention pain scores were lacking, this study describes the overall effect of analgesic therapy on pain scores. Another limitation of our study is that the COMFORTneo score has been validated in preterm infants, but not specifically in NEC patients.^28^ Unfortunately, other scores for assessing prolonged pain, such as the EDIN scale (Échelle Douleur Inconfort Nouveau-Né) and the N-PASS scale (Neonatal Pain, Agitation and Sedation Scale), have not been validated specifically in NEC patients either.^31,32^ The validation studies of the COMFORTneo, EDIN, and N-PASS score all included too few NEC patients to assess validity in this group specifically.^28,31,32^ More research is needed to determine the validity of prolonged pain scores in NEC patients.

The COMFORTneo score might be less reliable in NEC patients, since NEC patients are often severely ill and therefore less reactive showing a blank face and few movements, which could result in a normal or low COMFORTneo score in presence of pain.^31^ Therefore, the NRS pain and NRS distress score were evaluated as well, which represent nurses’ subjective estimates of pain and distress. We found a poor positive percent agreement between the COMFORTneo score and the NRS pain and NRS distress score, with the COMFORTneo score indicating pain more frequently than the NRS scores. This contradicts the results from a previous validation study of the COMFORTneo score by Van Dijk et al., which found a good agreement between the COMFORTneo score and the NRS pain and NRS distress score.^28^ The low positive percent agreement compared with the negative percent agreement might reflect the difficulty of detecting pain in NEC patients. Perhaps a different approach is needed, which integrates pain scores and other clinical parameters such as heart rate. A bedside pain dashboard, as has been suggested by Vinks et al., might enable the integration of different sources of information regarding a patient’s real-time pain level and trends and might benefit the implementation of personalized pain management.^33^ Current pain scores being repeatedly outside the normal window, suggest that analgesic therapy was not adequately tailored to the individual patient’s needs. Furthermore, pain persisting for several hours suggests suboptimal adherence to the local pain guideline, which prescribes considering extra medication in case of a high pain score and reassessing the pain score within an hour. Pain measurements being missed could have resulted in delayed analgesic therapy and thereby higher pain scores.

The finding that most NEC patients experience pain is of clinical importance, since there is evidence that neonatal exposure to pain could have negative long-term consequences. For instance, rodent studies have shown that exposure to pain during the neonatal period causes neurodegeneration and has negative effects on adult behavior.^7–9^ In humans, exposure to procedural pain during NICU admission has been associated with impaired brain development and cognitive development and with altered pain sensitivity later in life.^15,16,19^ The long-term consequences of intense visceral pain, as is caused by NEC, are largely unknown. It has been shown that NEC survivors have a lower pain tolerance and pain threshold at adult age, which might be explained by their exposure to pain during the disease.^26^

Most NEC patients in our study were treated with opioids, which may also harm neurodevelopment. Studies in rodents have shown that neonatal exposure to morphine in absence of pain causes neuronal damage and learning impairment, while exposure to morphine in presence of pain does not.^8–10^ Thus, in rodents, both neonatal pain and neonatal opioid exposure solely could be harmful to the developing brain, but in the presence of pain, exposure to opioids could have a neuroprotective effect. Conflicting effects of neonatal opioid exposure have been found in humans regarding brain development and cognition.^5^ These conflicting results might reflect differences in the balance between exposure to pain and analgesics.

Thus, it is important to find the right balance in the pain management of NEC patients: on the one hand, pain should be prevented by adequate analgesic therapy and on the other hand, excessive exposure to opioids should be prevented. NEC patients’ pain level should be measured regularly, and physicians should pay attention to pain scores and adequately adapt the analgesic therapy based on the pain score.

## Conclusion

Despite the use of acetaminophen and opioids, most NEC patients still experienced pain at some point. The analgesic therapy regimen often failed to relieve pain after a high pain score, resulting in pain persisting for hours in some patients. There is a lack of pain management guidelines for NEC patients. More research is needed to determine how the current analgesic therapy regimen can be adapted to improve the prevention and treatment of pain in NEC patients.

## Supplementary information

Supplementary Material
