# Supplementary material for: Pain management for necrotizing enterocolitis: getting the balance right
Source: Pediatr Res. 2022 Feb 15;92(5):1423–31. doi: 10.1038/s41390-022-01968-2 (PMC9700516; doi:10.1038/s41390-022-01968-2)
Supplement: Supplementary file 1 — Supplementary Material [file 41390_2022_1968_MOESM1_ESM.pdf]

**Supplement 1. COMFORTneo scale**

***Alertness***

1. Quiet sleep (eyes closed, no facial movement)
2. Active sleep (eyes closed, facial movement)
3. Quietly awake (eyes open, no facial movement)
4. Actively awake (eyes open, facial movement)
5. Awake and hyperalert

***Calmness/agitation***

1. Calm (appears lucid and serene)
2. Slightly anxious (shows slight anxiety)
3. Anxious (appears agitated but remains in control)
4. Very anxious (appears very agitated, just able to control)
5. Panicky (severe distress with loss of control)

***Respiratory response (only in mechanically ventilated children)***

1. No spontaneous respiration
2. Spontaneous respiration on ventilator
3. Unrest or resistance to ventilator
4. Actively breathes against ventilator or coughs regularly
5. Fights ventilator

***Crying (only in spontaneously breathing children)***

1. No crying
2. Faint crying
3. Soft crying or moaning
4. Hard crying
5. Intense crying or screaming

***Body movement***

1. No or minimal movement
2. Up to three slight arm and/or leg movements
3. More than three slight arm and/or leg movements
4. Up to three vigorous arm and/or leg movements
5. More than three vigorous arm and/or leg movements

***Facial tension***

1. Facial muscles fully relaxed, relaxed open mouth
2. Normal facial tension
3. Intermittent eye squeeze and brow furrow

4. Continuous eye squeeze and brow furrow
5. Facial muscles contorted and grimacing (eye squeeze, brow furrow, open mouth, nasal-labial lines)

***(Body) muscle tone*** ***(observation only)***

1. Muscles fully relaxed (open hands, dribbling, open mouth)
2. Reduced muscle tone; less resistance than normal
3. Normal muscle tone
4. Increased muscle tone (clenched hands and/or clenched, bent toes)
5. Extreme muscle tone (rigidity and flexion of fingers and/or toes)

**Supplement 2. Erasmus MC – Sophia Children's Hospital NICU Pain protocol (shortened and translated version)**

*Necrotizing enterocolitis*

Necrotizing enterocolitis (NEC) is generally a very painful condition. Analysis of our own population has shown that both pre- and postoperatively morphine doses of 10 to 15 microgram/kg/h and often fentanyl are needed to achieve adequate COMFORTneo scores. It is essential to regularly assess the level of pain. Few movements, a blank face and lying remarkably still are often seen as a sign of pain in NEC patients. In NEC patients, it is recommended to administer the combination of morphine and acetaminophen as standard treatment. The reason for this is that less morphine might be required if acetaminophen is administered as well.[1] Before nursing/painful moments, intermittent fentanyl may be added. If a patient is experiencing unacceptable pain despite maximum dosing of continuous morphine (20 microgram/kg/h) and intermittent acetaminophen and fentanyl boluses around nursing/interventions, morphine should be discontinued and switched to continuous fentanyl. If this is not desirable, a low dose of midazolam can be initiated instead. Midazolam and morphine act synergistically.[2]

*Pain assessment*

The COMFORTneo scale is a pain measurement instrument that has been validated at the Neonatology department of the Sophia Children's Hospital and that is since being used worldwide. A neonate's level of pain is assessed by observation by a nurse or physician. A COMFORTneo score ≥ 14 is regarded too high. A score ≤ 8 is, if sedatives or analgesics are administered, regarded as oversedation. In recent years, nurses assessed the COMFORTneo score in every patient during every shift.[3] Our own analysis has shown that in 90% of patients the COMFORTneo score was indeed assessed every shift. In 84% of COMFORTneo score assessments, the patient was comfortable. In case of a too high COMFORTneo score, which indicates that the patient is in pain, treatment was given in only 40% and re-assessment occurred in 31%. Also in case of oversedation, treatment adaptation and re-assessment rarely occurred. Thus, in both cases improvement is necessary. The COMFORTneo score should always be assessed in combination with the NRS pain and NRS distress score. These scores represent a nurse's estimate of pain and distress with a score ranging from 0 (no pain or distress) to 10 (worst imaginable pain or distress). An NRS score ≥ 4 is regarded too high. The NRS scores aid in differentiating between pain and distress.

Indications for pain assessment:

- Once every shift in every NICU patient

- When pain or distress is suspected
- When oversedation is suspected
- Before initiating analgesic therapy and 0.5 (in case of fentanyl) to 1 hour after initiation of analgesic therapy
- If continuous or intermittent analgesic therapy is given, the COMFORTneo and NRS scores should be assessed twice per shift.
- Before a change (increase or decrease) in analgesic dosage and 1 hour afterwards

*Analgesic therapy*

Recommended dosing for intravenous acetaminophen

| **Current weight** | **Loading dose** | **Maintenance dose per day** | **Maintenance dose per bolus** |
|---|---|---|---|
| < 750 g | 12,5 mg/kg | 20 mg/kg/day | 5 mg/kg every 6 hours |
| 750-1500 g | 12,5 mg/kg | 24 mg/kg/day | 6 mg/kg every 6 hours |
| 1500-300 g | 15 mg/kg | 30 mg/kg/day | 7,5 mg/kg every 6 hours |
| 3000-5500 g | 15 mg/kg | 40 mg/kg/day | 10 mg/kg every 6 hours |

Recommended dosing for intravenous morphine

| **Current weight** | **Loading dose (ug/kg/30 min)** | **Initial maintenance dose (ug/kg/h)** | **Maximum maintenance dose (ug/kg/h)** |
|---|---|---|---|
| < 1500 g | 50 | 5 | 20 |
| > 1500 g | 100 | 10 | 20 |

Recommended dosing for intravenous fentanyl

| **Weight / age** | **Bolus dose (max 6 dd)** | **Continuous dose** |
|---|---|---|
| All similar | 0.5-3.0 ug/kg | 0.5-3.0 ug/kg/h |

*Decision tree*

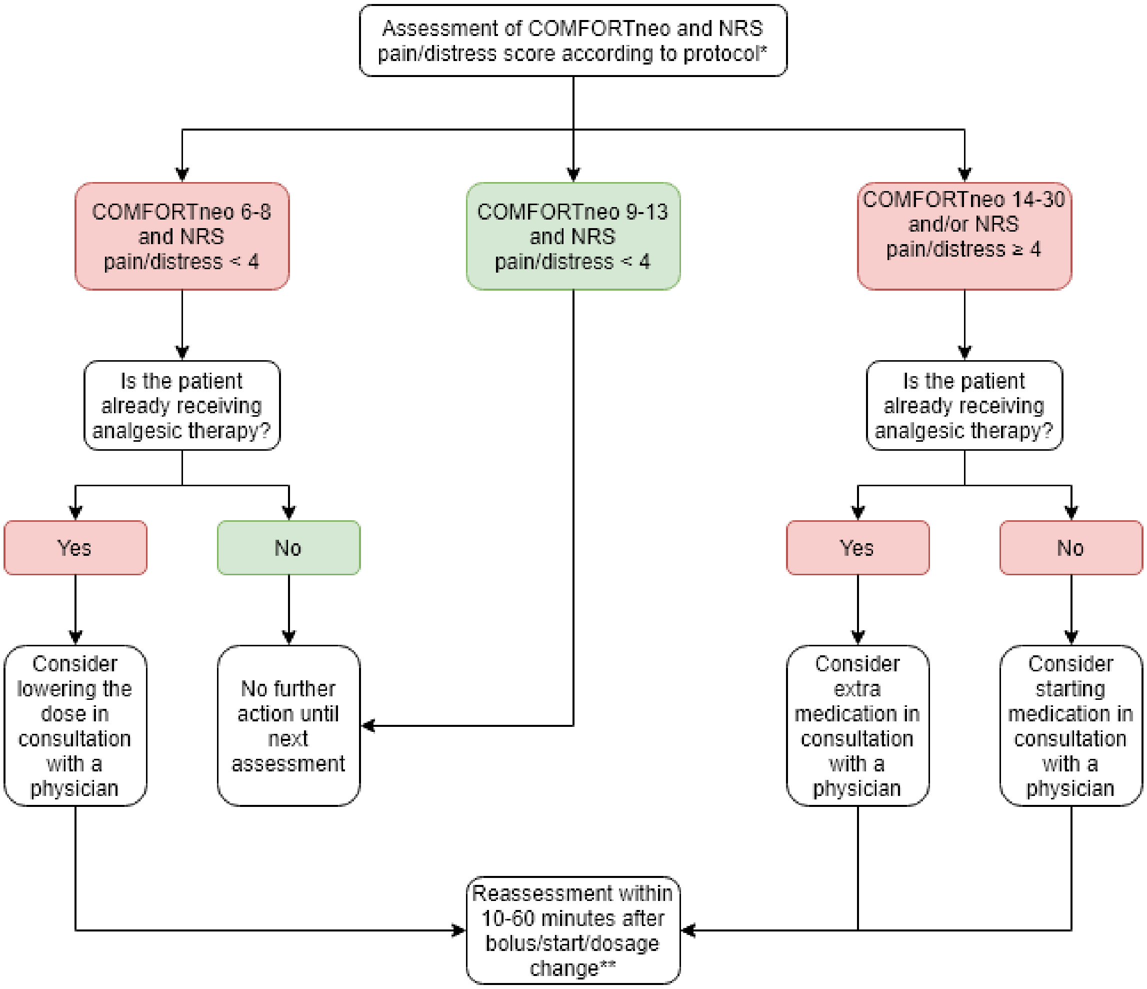


General guidelines

- The COMFORTneo score should always be assessed in combination with the NRS pain and NRS distress score
- In case of high scores also consider causes of pain/distress such as improper IV placement, sputum, medication, posture, hunger
- Consider options to offer comfort in every patient, for example via NIDCAP

*Guidelines pain assessment

- Standard 1 x per shift
- In case of analgesic therapy 2 x per shift
- On suspicion of pain
- On suspicion of oversedation
- Before and after starting/stopping analgesics

**Expected duration until effect (IV):

- Fentanyl: 2-3 min
- Morphine: 20 min
- Midazolam: 5-10 min
- Paracetamol: 5-10 min

*References*


1 Wong, I., St John-Green, C. & Walker, S. M. Opioid-Sparing Effects of Perioperative Paracetamol and Nonsteroidal Anti-Inflammatory Drugs (Nsaids) in Children. *Paediatr Anaesth* **23**, 475-495 (2013).

2 Hendrickx, J. F., Eger, E. I., 2nd, Sonner, J. M. & Shafer, S. L. Is Synergy the Rule? A Review of Anesthetic Interactions Producing Hypnosis and Immobility. *Anesth Analg* **107**, 494-506 (2008).

3 Aukes, D. I., Roofthooft, D. W. E., Simons, S. H. P., Tibboel, D. & van Dijk, M. Pain Management in Neonatal Intensive Care: Evaluation of the Compliance with Guidelines. *Clin J Pain* **31**, 830-835 (2015).
